# The advantages and limitations of trait analysis with GWAS: a review

**DOI:** 10.1186/1746-4811-9-29

**Published:** 2013-07-22

**Authors:** Arthur Korte, Ashley Farlow

**Affiliations:** 1 Gregor Mendel Institute of Molecular Plant Biology, Vienna, Austria

**Keywords:** GWAS, *Arabidopsis*, Mixed model, Effect size, Genetic heterogeneity

## Abstract

Over the last 10 years, high-density SNP arrays and DNA re-sequencing have illuminated the majority of the genotypic space for a number of organisms, including humans, maize, rice and *Arabidopsis*. For any researcher willing to define and score a phenotype across many individuals, Genome Wide Association Studies (GWAS) present a powerful tool to reconnect this trait back to its underlying genetics. In this review we discuss the biological and statistical considerations that underpin a successful analysis or otherwise. The relevance of biological factors including effect size, sample size, genetic heterogeneity, genomic confounding, linkage disequilibrium and spurious association, and statistical tools to account for these are presented. GWAS can offer a valuable first insight into trait architecture or candidate loci for subsequent validation.

## 

The causal relationship between genetic polymorphism within a species and the phenotypic differences observed between individuals is of fundamental biological interest. The ability to predict genetic risk factors for human disease and agronomically important traits like growth rate and yield in plants require an understanding of both the specific loci that underlie a phenotype, and the genetic architecture of a trait. This relationship between phenotype and genotype has been of major interest at least since Mendel postulated the existence of ‘internal factors’ that are passed on to the next generation.

Forward genetics, in which many individuals that differ in genotype are screened for phenotypes of interest, has been a hugely powerful tool to address such questions. In general, the raw genetic differences being screened are obtained either by mutagenesis or sampled from a natural population. Any phenotypic differences identified are connected back to the underlying causative loci via various mapping approaches including Quantitative Trait Locus (QTL) mapping. In this perspective we consider a complementary and powerful tool for connecting the genotype-phenotype map, Genome-Wide Association Studies (GWAS).

QTL mapping has proved, and remains, a powerful method to identify regions of the genome that co-segregate with a given trait either in F2 populations or Recombinant Inbred Line (RIL) families. The key components of the flowering time pathway in *Arabidopsis* have been dissected in this way [1-3]; for a review of natural variation and QTL mapping in *Arabidopsis* see [4]. Despite this success, QTL mapping suffers from two fundamental limitations; only allelic diversity that segregates between the parents of the particular F2 cross or within the RIL population can be assayed [5], and second, the amount of recombination that occurs during the creation of the RIL population places a limit on the mapping resolution. Resolution can be dramatically improved with several generations of intercrossing when establishing the RIL population, e.g. advanced intercross RILs [6]. Meanwhile, allelic diversity within a mapping population can be increased (up to a point) by intercrossing multiple genetically diverse accessions before establishing the RILs, e.g. the Multi-parent Advanced Generation Inter-Cross (MAGIC) and *Arabidopsis* multi-parent RIL (AMPRIL) [7,8].

Nevertheless, the allele frequencies and combinations present in any such lab population will differ from those in the natural population [9]. For many applications this does not present a problem, but it does confound the analysis of epistasis for example, and offers only a limited view of the functional diversity present within the natural population.

GWAS overcome the two main limitations of QTL analysis mentioned above, but introduce several other drawbacks as a trade-off (discussed below). Generally, after identifying a phenotype of interest, GWAS can serve as a foundation experiment by providing insights into the genetic architecture of the trait, allowing informed choice of parents for QTL analysis, and suggesting candidates for mutagenesis and transgenics. Thus, GWAS are often complementary to QTL mapping and, when conducted together, they mitigate each other’s limitations [10,11].

The basic approach in GWAS is to evaluate the association between each genotyped marker and a phenotype of interest that has been scored across a large number of individuals. This approach was pioneered nearly ten years ago in human genetics [12], with nearly 1,500 published human GWAS to date [13]. GWAS are now routinely applied in a range of model organisms including *Arabidopsis*[14] and mouse [15], and to non-model systems including crops [16-18] and cattle [19].

In this review we will discuss the advantages and limitations of running a GWAS in *Arabidopsis*, issues that are generally relevant to other organisms. We consider sample size and mapping panel composition, statistical approaches to overcome genetic confounding and methods to identify and account for complex genetic architectures.

### Self-fertilisation makes *Arabidopsis* particularly well suited to GWAS

*Arabidopsis* thaliana has proved an almost ideal organism in which to conduct GWAS because it can be maintained as inbred lines via continued self-fertilization, thus it is possible to repeatedly phenotype genetically identical individuals. Because more than 1,300 distinct accessions have been genotyped for 250,000 SNPs [20] all a researcher requires is the phenotype of several hundred lines for a trait of interest. In addition to the landmark proof-of-concept GWAS study of 107 phenotypes [14], numerous other traits including glucosinolate levels [21], shade avoidance [22], heavy metal [23] and salt tolerance [24], flowering time [25], and other life history traits [26] have been successfully analyzed.

Importantly, major improvements in the statistical methodology have occurred recently, including the use of mixed models that take into account the confounding effect of genetic background. This has been implemented via various R and Python packages, or as a first point of call, one can make use of the online tool: http://gwas.gmi.oeaw.ac.at[27]. This web application comes preloaded with the genotype data for all commonly used accessions, provides several statistical options, and facilitates a meta-analysis across published traits. Whereas several years ago, a complete genome-wide scan of a few hundred individuals could easily take a day, a simple single marker scan (termed a marginal test, ignoring epistasis and other interactions) of a few hundred thousand SNPs runs on a PC or the web-based application in a few minutes.

### Genetic architecture; rare variants of large effect, or common variants of small effect?

The motivation to conduct GWAS can be either to identify causative/predictive factors for a given trait, or to determine aspects of the genetic architecture of the trait (i.e. the number of loci that contribute and their respective contribution to the phenotype). Some traits are underpinned by a small number of loci with large effect sizes (a simple genetic architecture) and are highly amenable to GWAS. This scenario might be common for traits under biotic selection [28]. Other traits may possess more complex architectures that present difficulties for GWAS. Two possibilities are either that a trait is controlled by many rare variants, each having a large effect on the phenotype, or in contrast, many common variants of only a small phenotypic effect. In both cases the causative variants may be clustered in one or a small number of genes, or across many genes (polygenetic).

The power of GWAS to identify a true association between a SNP and trait is dependent on the phenotypic variance within the population explained by the SNP (Figure 1a). The phenotypic variance is determined by how strongly the two allelic variants differ in their phenotypic effect (the effect size), and their frequency in the sample. Because of this both rare variants and small effect size present problems for GWAS [29,30].

**Figure 1:**
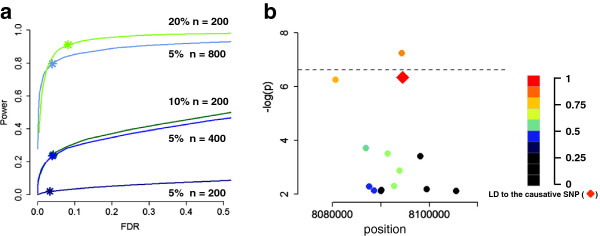
**Sample size and effect size. a)** Power and FDR for an idealized phenotype. Simulations in which a single random SNP explaining 5%, 10% or 20% of the phenotypic variance (with heritability ~0.75) were performed in either 200, 400 or 800 individuals [67]. Simulations are based on the available SNP data for *Arabidopsis*[20], with structure added by giving 10,000 random SNPs a tiny effects size. The star indicates power (the ability to find true positives) and FDR (false positives) at the 5% bonferroni-corrected threshold for 220,000 markers. **b)** An example of one particular simulation in which the causative SNP (red diamond) is not the most significant SNP in the local window. Remaining SNPs are colored according to their linkage to the causative SNP. Dashed line denotes the 5% bonferroni-corrected threshold for 220,000 markers.

Additionally, rare variants suffer from being in strong or complete association with many other non-causative rare variants within the genome, regardless of the LD decay, and thus a single causative locus may drag with it many synthetic associations [31]. This point is illustrated clearly if one considers multiple private SNPs within an individual: they are completely linked regardless of their genomic locations.

How does one increase the power to detect meaningful association when variants are either at low frequency or have a small effect size? Several important considerations including sample size, incomplete genotyping, genetic heterogeneity and accounting for confounding genetic background are discussed below. We note however, that the importance of rare variants for a particular trait may also be disentangled using QTL analysis as rare variants are elevated to intermediate frequency by the crossing scheme.

### Sample size and genetic heterogeneity: how to choose your mapping panel?

To date, most analyses performed with *Arabidopsis* have used only a few hundred individuals, but for some traits, meaningful results can be obtained with less than 100 accessions [14]. This suggests that the traits considered were underpinned by only a few loci that explain a large portion of the phenotypic variance. The situation looks different in humans, where typically a large number of small effect loci are found and most analyses require several thousand individuals to detect these [32,33]. Genetic architectures with many small effects are observed in other animals [34] or maize [35]. It remains to be seen whether there is a general trend for different genetic architectures between outcrossing and selfing species. On the other hand, human disease states may in fact be a special class of traits driven by numerous small effect deleterious mutations, whereas, loci with intermediate effect size have been shown to underlie traits such as human eye and skin-colour [36,37].

Despite the success of GWAS in *Arabidopsis*, many traits will be polygenic with small effect size; hence, increasing the sample size will improve the power to recover meaningful associations (Figure 1a). Given this, how does one select a mapping panel? One approach is to use a star-like design by including geographically distant accessions. This will maximize the genetic variance within the sample [25], but has the potential to introduce genetic heterogeneity. For reasons including local adaptation, different variants may underlie a trait in samples collected from different locations [26]. This genetic heterogeneity will reduce the power to recover either variant, because it weakens the correlation between the phenotype and any specific variant (Figure 2).

**Figure 2:**
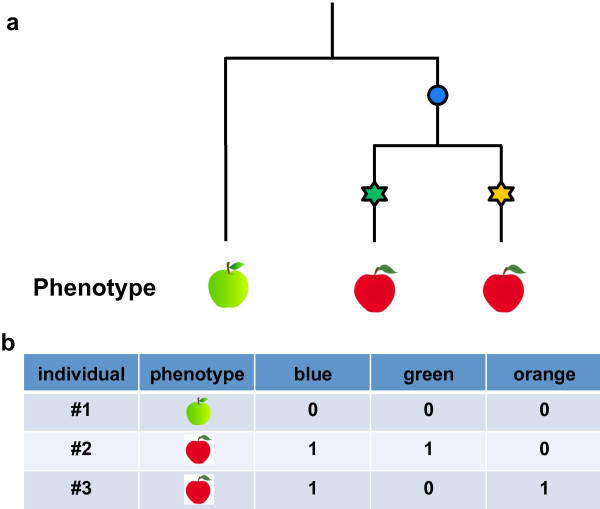
**Synthetic association due to genetic heterogeneity. a)** A theoretical phylogenetic tree of three individuals upon which three mutations occur. The two most recent mutations (stars) cause a change in phenotype (red fruit). **b)** The older blue mutation has no affect on fruit colour, but is in perfect correlation with the trait. Neither causative mutation are very good predictors of the phenotype.

Interestingly, genetic heterogeneity can lead to a non-causative marker being a better descriptor of the phenotype than a causative one [38]. Consider the case of two recent, rare mutations that both influence the same phenotype: any marker linked with both alleles will, despite being non-causative, show stronger association with the phenotype than each of the two single markers alone (Figure 2). Such synthetic associations, while false positives in the sense that they do not cause the phenotype, still prove valuable, as they are all one needs to predict the phenotype.

It becomes possible to disentangle the contribution of genetic heterogeneity by including ‘competing’ variants as cofactors within a mixed model setting [39]. If in fact, the most significant SNP is the sole causative marker, including it as a cofactor should account for all the phenotypic variance contributed from that genomic region. By fitting multiple SNPs into the mixed model, one can potentially disentangle the minimal number of SNPs underlying a distinct GWAS peak. Identifying the set of SNPs for inclusion in such a model is however, non-trivial, and can lead to over-fitting.

A second approach to increase sample size is to densely sample a local population that shows phenotypic diversity. This has the potential advantage of minimizing genetic heterogeneity, but the draw back that variants relevant to global phenotypic diversity may remain at low allele frequency or absent completely.

However, increasing the sample size may not always resolve a rare-variant architecture. One proposed solution is to collapse several SNPs in a region into a single indicator variable and use this as a composite genotype [40]. One could imagine this as a way of simplifying a highly complex pattern of variation into only two haplotypes. Unfortunately, the rational of how to collapse SNPs is non-trivial [41-43].

### An imperfect genotype

It is noteworthy that causal variant(s) for most phenotypes are unlikely to be present in currently available array-based SNP datasets. The 250 K SNPs in *Arabidopsis* represent only a few percent of the SNPs that are segregating within the population. Recent whole-genome sequencing has revealed a much higher SNPs density in *Arabidopsis*[44-46], with approximately 7 Million SNPs within a worldwide sample. Despite this, significant associations are detectable because causative variants (be they SNPs or structural variants) are often in sufficient linkage disequilibrium (LD) with genotyped markers. In *Arabidopsis*, LD generally decays 50% within 5 Kb [45]; hence, the 250 K SNPs (on average one SNP every 600 bp) tag almost all of the non-repetitive genome, and thus enable GWAS [14,47].

In the near future the ‘full’ genome sequence of more than 1,000 accessions will become available (http://www.1001genomes.org). This set of ‘all’ SNPs, structural variant, copy number and transposable element variation will presumably include most causative variants. It is noteworthy, that in principle any of these genotypes can be used for GWAS.

Will the inclusion of such full sequence actually prove helpful? No matter how many variants are included, the LD structure of the data and the unusual occurrence of long-range LD observed between SNPs within (and sometimes between) *Arabidopsis* chromosomes [46] will always make the disentanglement of causative variants from linked neutral markers difficult. However, any drawbacks caused by this LD structure will be strongly outweighed by the benefits gained by knowing about all variants during subsequent hypothesis testing and follow up studies.

Missing or low quality data is a major issue for both SNP chips and re-sequencing datasets. Excluding poorly genotyped variants from only a subset of individuals introduces an unequal sample size across sites, making the downstream statistics more complex. Commonly, this is overcome via the imputation of missing data [48], in which the state of an un-genotyped marker is inferred from the haplotypes of the other individuals. This approach may be valid when data is missing due to technical reasons (low coverage sequencing or poor hybridization to genotyping arrays); however, it is likely to miss-infer the correct state if more than two alleles are present at a site, which will occur whenever SVs and CNVs overlap a SNP. Alternatively, one may allow uncertainty in the genotype [49] by calculating a probability score for each SNP, which is then used to weight the regression.

### Confounding due to relatedness

Two major issues discussed above: that related individuals share both causal and non-causal alleles, and that LD between these sites can lead to synthetic associations, are actually a single problem, that of confounding due to genetic background [50]. A powerful method to account for this artifact was first developed in the field of animal breeding: mixed models that handle population structure by accounting for the amount of phenotypic covariance that is due to genetic relatedness (i.e. including relationship or kinship as a random term within the model). Since then, mixed models have been applied to GWAS [11,51-53], and can markedly reduce the number of false positive associations (Figure 3).

**Figure 3:**
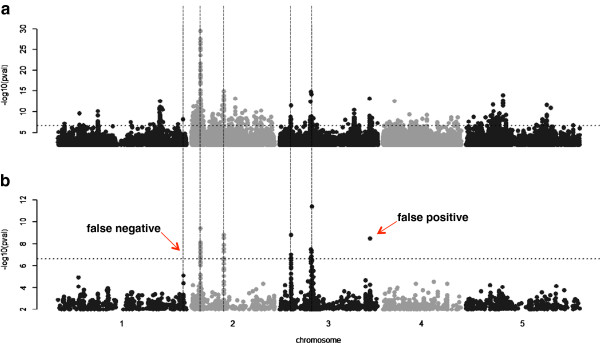
**Taking genetic background into account improves the performance of GWAS.** Manhattan plots for a simulated trait, in which each data point represents a genotyped SNP, ordered across the five chromosomes of *Arabidopsis*. Five SNPs (indicated by vertical dashed lines) were randomly chosen to be ‘causative’ and account for up to 10% of the phenotypic variance each. GWAS using **a)** a linear model, and **b)** a mixed model that accounts for population structure and other background genomic factors. The simple linear model leads to heavily inflated p-values and the five causative markers are not the strongest associations. The mixed model is superior, but still leads to one false negative and one false positive. A dashed horizontal line denotes the 5% Bonferroni threshold.

Unfortunately, any relationship matrix used to correct for population structure can only serve as a proxy for the real underlying genetic background [50]. Intuitively, one only wished to correct for confounding markers that are associated with the trait of interest. One approach is to only include SNPs in the relationship matrix that show the strongest linear correlation with the trait [54].

### Judging the outcome

On what criteria can one judge the most appropriate GWAS method for a particular trait? The most basic and often informative approach is a correction for multiple testing (usually a 5% Bonferroni threshold is used) and inspection of Q-Q plots and Manhattan plots for evidence of *P* value inflation (Figures 3 and 4). Both approaches give a general impression of the data, i.e. are there too many, or too few significant SNPs relative to ones prior expectation? The main limitation of these corrections is the assumption that every SNP tested is independent. Structure in the *Arabidopsis* population clearly violates this assumption and thus many spurious associations survive a multiple testing correction due to LD in the data.

**Figure 4:**
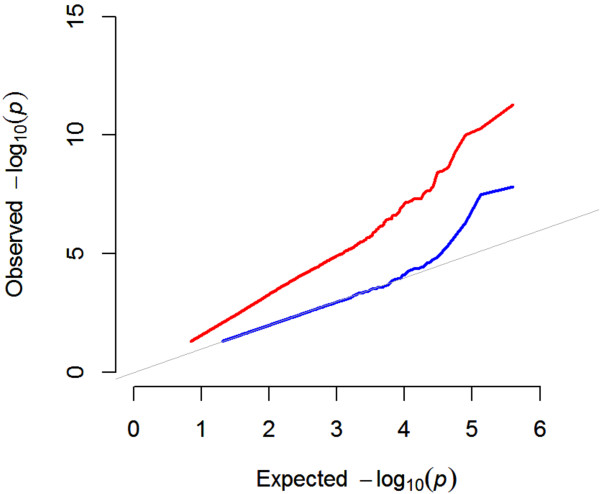
**The mixed model dramatically reduces inflation of *p*-values.** Quantile-Quantile plot showing strong p-values inflation for a marginal GWAS that does not consider population structure (red line). Accounting for population structure with the mixed model dramatically reduces inflation (blue line). The grey line indicates the expected *p*-value distribution under the null hypothesis of no causative markers in the data. Note, that after correction for population structure, only the most significant markers deviate from the null expectation.

The most informative criterion of performance is the proportion of false positive and false negative associations in a simulated dataset (that maintains the LD characteristics of the real population), typically expressed as false discovery rate (FDR) and power (Figure 1). Given the aims of the study, one may consider a high FDR for some projects (e.g. investigating the genetic architecture of a trait) and a low FDR for others (e.g. identifying candidate loci for follow-up studies).

### Adding it all up: heritability

Narrow sense heritability is a measure of the contribution of additive genetic variants to the observed phenotypic variance; this can be thought of as how strongly the phenotype is connected to the genotype. The mixed model, used to run GWAS, partitions the observed phenotypic variance into additive genetic and non-genetic components. These estimates can (under the assumption of the infinitesimal model) be used to calculate heritability, usually referred to as pseudo-heritability.

For some traits (flowering time in *Arabidopsis* for example) the pseudo-heritability may actually exceed heritability estimated from replicates (A. Korte personal observation). However, for many traits (especially in humans) the pseudo-heritability is much lower. This could be thought of as a special case of missing heritability. Missing heritability normally refers to the portion of genetic variance that cannot be explained by all significant SNPs [32]. This discrepancy might partly result from incomplete linkage between causative variants and those genotyped, or due to rare variants [31] (however, see [55]). The problem then is a fundamental limitation of GWAS to identify variants of small effect, or viewed another way, a limitation of running GWAS in a small and heterogeneous sample. Inclusion of full sequence data and increased sample size could in theory overcome this issue.

Furthermore, it has been suggested that the epigenetic state might also contribute to heritability in *Arabidopsis*[56,57]. Given that a variable epigenetic state might modulate the connection between SNP and trait it would be appropriate to consider this intermediate biological information. This might be implemented with the integration of genetic, epigenetic, gene expression and phenotype information into a joint model. Estimating the parameters of such a model would require a substantial sample size.

### Accounting for interactions within the genome and the environment

Marginal GWAS do not consider the genetic interaction between loci (epistasis), or the interaction between loci and the environment. Epistatic interactions between genes poses a problem to association mapping, yet are likely to make a major contribution to the *Arabidopsis* phenotype [58].

Although strategies for identifying epistatic interaction in GWAS have been proposed [59-61], complete genome-wide interaction scans suffer (if not computationally, at least statistically) from the massive number of tests that need to be performed. The computational problems could be overcome using graphics processing units (GPUs) [62].

Identifying meaningful associations from the trillions of pair-wise tests is a serious challenge. Various approaches to reduce the number of tests consider only loci previously shown to be important in the marginal GWAS or make use of dimension reduction [63,64]. Incorporating the *Arabidopsis* Interactome data [65] is another possibility. As an example, by taking known network topologies into account and testing specific models on a case-by-case basis, a recent study used the well-characterized glucosinolate pathway and combined it with GWAS to identify new loci that are sensitive to environmental fluctuations [21].

The contribution of a gene to a trait may vary depending on the environmental conditions, and methods to identify such gene-by-environment (GxE) interactions have been suggested [66]. In this setting, the ability to repetitively phenotype the same genotype (due to selfing) allows one to test associations in several different environments (e.g. flowering time at two temperatures or fitness at different locations). Statistical models that analyze correlated traits (one can consider a trait measured in two environments as two correlated traits), while still correcting for population structure, have been proposed [67]. This allows detection of previously undetected associations and the decomposition of effects into genetic and environmental components, shedding light on trait architecture [67,68]. Interestingly, this method is more powerful at detecting GxE crossover effects, in which the effect of an allele is opposite in the different environments, and less powerful at identifying scaling effects, in which only the magnitude of the effect changes [67,69].

Phenotyping the same *Arabidopsis* line multiple times under controlled environmental conditions increases precision of the trait mean, but also allows one to estimate the phenotypic variance. Simulations suggest that selection affects variance-controlling loci even more strongly [70]. For this approach to work the extent to which a phenotype is buffered must vary between individuals, a likely situation. To date, most GWAS have considered only the trait mean, aiming to understand the genetic contribution to a particular phenotype *per se*. However, by considering the phenotypic variance it becomes possible to uncover the genetic basis of robustness and plasticity.

It is interesting to consider that the most dramatic environmental change an allele might experience is a shift into a different genetic background. While technically any difference that results is the outcome of epistasis (gene-by-gene interactions) one can essentially model this as a GxE effect.

### Looking forward

GWAS methodology has advanced such that it is now a powerful tool for the analysis of simple traits under additive genetic scenarios, and for the dissection of more complex genetic architectures. Many phenotypes of interest in humans and plants are highly quantitative, and as such GWAS may fail to uncover the causative loci we seek. One possible solution is to refine the phenotype of interest by scoring a trait more proximal to the underlying genetics [71]. This has the potential to reduce the number of loci that contribute to the trait and thus increase the power to detect them.

It is an important consideration (or limitation) that even under the simple simulation scenario of a single causative locus with high heritability presented in Figure 1b, the most significant SNP is not always the true causative locus. Such a synthetic association is a natural consequence of the linkage and error structure of the data, and thus may persist despite an increase in the sample size.

The literature now contains numerous examples of GWAS that uncover the underlying genetics. Still, missing genotypes, genetic heterogeneity, unexpected LD, small effects size, low allele frequency or complex genetic architectures remain a challenge. The collection of GWAS methods to account for such factors will continue to grow. However, the best predictors of success will remain a well-defined trait, an appropriate statistical model and finally, the validation of candidates.

### Glossary

*Effect size* The average phenotypic difference of two alleles at a locus.

*Genetic architecture* The network of genetic variants that underlie a given trait, including the number, effect size, and allele frequency of causative alleles, and all additive and epistatic interactions between them.

*Genetic background* All loci that do not contribute to a given trait in a particular environment. Factors including population structure can cause partial correlation between the genetic background and a trait.

*Genetic heterogeneity* When different loci, either within a single gene (allelic heterogeneity) or in different genes (genic/locus heterogeneity), produce the same phenotypic effect in separate individuals.

*Gene-by-Environment (GxE) interaction* When the phenotypic effect of a locus is different in distinct environments (see [72]).

*Gene-by-Gene (GxG) interaction or Epistasis* The non-additive interaction of two or more loci (see [73]). Allelic combinations between sites may result in a higher (positive epistasis) or lower (negative epistasis) phenotype than expected from the effect size at each locus alone.

*Heritability* The proportion of phenotypic variance attributed to variance in genotype (broad sense heritability) in a particular environment (see [74]). The contribution from additive genetic variants (i.e. excluding dominance and epistasis) is the narrow sense heritability (or breeding value) which can be estimated from the regression of offspring phenotypic values on parental phenotypes.

*Linkage disequilibrium (LD)* The non-random co-occurrence of two or more alleles. LD naturally occurs between loci in close proximity, and is broken down by recombination. Higher than expected LD can be maintained, even across different chromosomes, by selection or population structure.

*Mixed models* A statistical model that contains both fixed and random effects, used to estimate correlations between phenotypes and genotypes, while taking into account the relatedness between individuals.

*Phenotypic variance* A measure of the spread of trait values within a population. Phenotypic variance results from genetic (see heritability) and environmental factors. The proportion of phenotypic variance explained by a single locus is a product of its effect size and allele frequency.

*Pseudo-heritability* An estimate of narrow sense heritability from the mixed model. This is the fraction of phenotypic variance that can be explained by the genetic relatedness between individuals (as estimated by a genome-wide kinship matrix from SNP data).

*Synthetic association* The association of a non-causative marker with a given trait, driven by linkage to one or more causative markers and/or an unmeasured source of error.

### Competing interest

The authors declare that they have no competing interests.

### Authors‘ contribution

AK and AF wrote and edited the manuscript together. Both authors read and approved the final manuscript.
